# Factors Influencing Operational Delays in Prehospital Trauma Interventions: Evidence from a Romanian Cohort

**DOI:** 10.3390/medicina62091632

**Published:** 2026-08-25

**Authors:** Alexandra Haută, Radu-Alexandru Iacobescu, Paul Lucian Nedelea, Mihaela Corlade-Andrei, Teofil Blaga, Marius Ivanuta, Ana Ivanuta, Carmen Diana Cimpoeșu

**Affiliations:** 1 Gigore T. Popa University of Medicine and Pharmacy, 700115 Iasi, Romania; alexandra.hauta@umfiasi.ro (A.H.); paul.nedelea@yahoo.com (P.L.N.); corladeandrei.mihaela@yahoo.com (M.C.-A.); teofil_blaga@umfiasi.ro (T.B.); marius.ivanuta@umfiasi.ro (M.I.); ion.ana-maria@d.umfiasi.ro (A.I.); dcimpoiesu@yahoo.com (C.D.C.); 2 Emergency Department, “Sf. Spiridon” County Clinical Emergency Hospital, 700111 Iasi, Romania

**Keywords:** prehospital trauma care, emergency medical services, operational delays, on-scene time, time factors

## Abstract

*Background and Objectives*: Trauma is a frequent cause of emergency healthcare requests that requires timely intervention and transport to definitive care, ideally within the first 60 min. On-scene time is considered a modifiable component of the prehospital phase and holds the greatest potential for optimizing prehospital operational time. However, factors contributing to on-scene delays are emerging across different trauma systems, highlighting the need to optimize operational efforts. Data on these factors vary across settings and have rarely been explored in Europe. Romania is an Eastern European country with a distinct demographic distribution and a unique emergency healthcare system. This study aims to evaluate compliance with the golden hour of prehospital trauma care and factors associated with on-scene and total prehospital operational time in Iasi County, Romania. *Materials and Methods*: A retrospective analysis of data from the electronic dispatch registry from Iasi County was performed for cases dating from January 2025 to February 2026. Data regarding demographics, trauma characteristics, and operational data were retrieved. Generalized linear modeling was used to investigate associations of total prehospital time or on-scene time with demographic and case-specific factors. Multivariable logistic regression was used to evaluate factors associated with total prehospital time exceeding 60 min and on-scene time of 20 min or more. Further sensitivity analyses were performed to account for scene complexity. Age association with operational time was assessed for non-linearity using restricted cubic splines. *Results*: Of the 8553 included trauma cases, 33.1% presented with on-scene delays, and 59.5% presented with total prehospital time above 60 min. Factors associated with on-scene time were age, rural environment, nature of trauma and severity, and scene-related complexity factors such as number of performed interventions and multiple victim incidents, while for total prehospital time the moment of intervention was also a relevant factor. Age displayed a non-linear association with on-scene time (*p* < 0.001), with a more pronounced increase after approximately 60 years of age, while for total prehospital time no evidence of non-linearity was observed (time ratio: 1.0024, 95% CI:1.0019–1.0029, *p* < 0.001). Rural environment was also a factor associated with both operational measurements (time ratio:1.17, 95%CI:1.15–1.2, *p* < 0.001 for on-scene time and time ratio:1.42, 95% CI:1.38–1.45, *p* < 0.001 for total prehospital time) and was associated with higher odds of on-scene time ≥ 20 min (OR:1.74, 95% CI:1.58–1.92, *p* < 0.001) and total prehospital time >60 min (OR:4.91, 95% CI:4.45–5.43, *p* < 0.001). *Conclusions*: The large proportion of cases identified with delayed operational times supports the need for further exploration of modifiable factors. The study highlights that, beyond case-specific factors and scene complexity, age and rural environment are relevant to prehospital operational time in Romanian trauma care settings. Further studies are needed to determine the underlying mechanisms of these associations and find targeted strategies to improve operational efficiency.

## 1. Introduction

In Europe, trauma is a major cause of death and disability, with injuries accounting for 20.2% of all deaths within the working population [1]. While trends for trauma-related deaths among the elderly population are significantly lower, studies in regions with developed emergency care systems showed that even lower-energy traumas carry significant consequences for elderly patients [2]. As such, every trauma-related emergency care is a potential critical event. Delivering optimal care in any trauma requires prompt recognition and intervention, as well as efficient coordination starting from the prehospital setting up to definitive care within the hospital.

The prehospital phase of the trauma response is a time-sensitive effort. Prehospital time has emerged as a critical factor affecting outcomes of injured patients [3]. Consequently, the golden hour has been empirically recognized as a critical cut-off for optimal prehospital trauma care [4]. The importance of this timeframe for reaching definitive care remains an active area of investigation [5]. Although findings remain heterogeneous across EMS models and geographic settings, on-scene time is becoming a more relevant metric of prehospital care performance [6]. The prehospital phase comprises multiple sequential components, including trauma activation, the response time from trauma call to on-scene arrival, the on-scene time until scene departure, and the transport time until patient handover to a nearby emergency hospital [7]. While response and transport times are more likely to depend on emergency system settings and infrastructure and are less likely to be modifiable, on-scene time has been extensively investigated as a means to reduce prehospital time [8]. This has led to the development of contrasting prehospital strategies, such as “stay-and-play” and “load-and-go,” in an effort to optimize care and outcomes [9]. So far, the evidence supporting the superiority of either strategy remains inconclusive, and current efforts focus on improving the on-scene operational performance of trauma responders. However, recent studies have found that several other factors contribute to on-scene delays beyond the rescue team’s care efforts. Frequently reported are demographic factors such as age, gender, and location (rural or urban), and trauma-related or intervention-related factors such as type of trauma, moment of injury (daytime or nighttime), and number of procedures performed [8,10,11,12,13]. Identifying these factors is important, as they can drive further protocol refinement for specific contexts and provide projections of care needs for at-risk groups. However, these factors have been explored in limited settings, which represents a missed opportunity warranting further investigation.

Data on factors influencing prehospital time and on-scene time have rarely been investigated in Europe. Most recently, Nilsbakken et al. performed a nationwide investigation in Norway and found remote settings as an important factor associated with increased prehospital time [7]. This was also the case in Sweden, where total prehospital time was higher in locations with a lower population density [14]. These studies investigated a limited number of factors in high-income settings and did not consider individual components of the prehospital phase. There continues to be a scarcity of data from mixed or resource-variable systems, including many European, middle-income, and less densely populated regions.

Romania is an Eastern European country with a mixed-model emergency medical system. The Romanian Emergency Medical System (EMS) is organized into regionally governed networks that consist of emergency dispatch, ambulance services, firefighter-rescue units, and hospital emergency departments, coordinated by the Department for Emergency Situations [15]. Following an emergency care request through the national 112 universal emergency number, all resources are dispatched through the integrated county communication centers. Prehospital trauma care is delivered by ambulance services (nurse-led teams or physician-led teams) or by the Mobile Emergency Service for Resuscitation and Extrication (SMURD), a physician-led team within the fire rescue services. These response capabilities are stratified by accessibility and injury severity and dispersed throughout the county based on projected resource requirements. This system was developed to provide rapid intervention, to ease interagency communication, and to align protocols with European emergency medicine practices [16]. Despite this system’s existence for over 30 years, compliance with the golden hour concept in contemporary practice and operational performance has not been explored. Furthermore, factors affecting prehospital time in trauma responses have not been considered. Given the substantial rural population and aging demographic that may represent distinct operational challenges for prehospital care, an assessment of these factors is warranted.

To our knowledge, no study has investigated operational prehospital trauma response intervals and their possible determinants in Eastern European countries. This study aims to characterize prehospital time intervals for trauma cases in Iasi County, Romania, and to evaluate factors associated with on-scene time and total prehospital time.

## 2. Materials and Methods

### 2.1. Study Design

This is a retrospective cohort study on trauma cases registered in the electronic dispatch registry of Iasi County, Romania, between January 2025 and February 2026. The evaluated timeframe was selected because it represents the most recent complete and relevant dataset available at the time of analysis. This study is reported in accordance with the Strengthening the Reporting of Observational Studies in Epidemiology (STROBE) guidelines, and the complete checklist can be consulted in Supplementary Table S1 [17].

All trauma-related emergency dispatches registered during the study period were screened for eligibility. This included road traffic injuries, head and neck trauma, burns and hypothermia, falls, drownings, electrocutions or other chemical burns, and violence-related trauma. All ages were eligible for selection. The cases were selected for analysis if they provided sufficient demographic data and had available time intervals for the prehospital phase. Cases were removed if, upon emergency service consultation, patients refused transport to the emergency service, chose an alternative transport route, or were deemed dead on arrival. Cases in which negative time was recorded for any of the components of the prehospital time, or exceeded 60 min for on-scene time without documented clinical or operational factors such as on-scene resuscitation, extrication, multiple-victim incidents, multiple on-scene treatments, or other special scene circumstances (difficulty accessing the victim, personnel safety issues) were also removed from the analysis. This was done to minimize the inclusion of potentially erroneous data caused by timestamp errors in the registry. All cases pooled from the registry were de-identified.

For each case, demographic data (gender and age) and case-specific data (time of care request, environment, type of trauma, triage code, type of rescue team dispatched, whether multiple victims were involved, and whether extrication was needed) were collected. No classification of intervention location was performed by the investigators. Data regarding environment (rural/urban) were retrieved as recorded by the responding emergency team. Triage severity was categorized according to the STAR (Simple Triage and Rapid Treatment) system and assigned by responding teams upon arrival on scene. The number of on-scene interventions was also recorded. The interventions were counted if they related to bleeding management, immobilization, airway management, or resuscitation.

### 2.2. Outcome Variables

Prehospital operational time was divided into three consecutive intervals: response time, on-scene time, and transport time. On-scene time was measured from scene arrival to departure for the nearest emergency hospital. Previously reported thresholds for acceptable on-scene time range from 10 to 30 min [8,12]. This wide range reflects the different approaches to on-scene trauma care. However, the literature also shows that an on-scene time of 20 min or greater is associated with increased mortality and unfavorable outcomes [18]. Given the literature-reported clinical significance, a value ≥20 min was considered prolonged on-scene time in this study. Total prehospital time is the interval from call receipt to patient handover and comprises all three phases. A cut-off value of 60 min was historically considered optimal for prehospital trauma care and was recently associated with unfavorable outcomes except mortality [19,20]. Consequently, a total prehospital time exceeding this cut-off was considered delayed. These time intervals were calculated from timestamps automatically recorded by the dispatch system when the emergency response teams updated operational status.

### 2.3. Statistical Analysis

All statistical analyses were performed using SPSS (version 23, IBM Corp., Armonk, NY, USA) and the Microsoft Excel Data Analysis ToolPak (version 2607, Microsoft Corporation, Redmond, WA, USA). The normality of continuous data was assessed using the Kolmogorov–Smirnov test. Between-group comparisons were performed using the Mann–Whitney U test or the Kruskal–Wallis test, with post hoc analysis using Dunn’s test with Bonferroni correction when appropriate. Correlations were assessed using Spearman’s rank test. For categorical variables, comparisons were carried out using the χ^2^ test.

To investigate potential factors associated with prehospital time intervals, two multivariable modeling approaches were conducted. First, on-scene time and total prehospital time were analyzed using a generalized linear model (GLM) approach. This approach treated the two outcome variables as continuous. Given the non-Gaussian, positively skewed distributions of the two outcome variables, the analysis assumed a gamma distribution and used the log-link function. Time ratios (TRs) relative to reference categories are reported, along with 95% CIs, after adjustment for other covariates.

Second, a multivariable logistic regression model was constructed for the dichotomized outcome variables on-scene time ≥ 20 min and total prehospital time > 60 min. Odds ratios (OR) and 95% CI were reported. Model fit was assessed using the Hosmer–Lemeshow goodness-of-fit test, and multicollinearity was assessed using Variance Inflation Factors (VIFs) and tolerance. Model performance and internal stability were assessed for the primary multivariable logistic models. For this purpose, ROC curve analyses, calculation of slope and model intercept from model estimates, and bootstrap resampling with 1000 bootstrap samples were performed.

Covariate selection was based on literature-reported relevance, plausible relationships with prehospital time intervals, and database availability. These included age, sex, intervention environment (urban/rural), moment of intervention, trauma type, triage category, type of team dispatched, and polytrauma status. Further sensitivity analysis was performed by adjusting for markers of scene complexity, such as the number of on-scene interventions, multiple-victim incidents, and extrication. These were omitted from the primary adjusted models to reduce the risk of overadjustment and reverse causation. The assumption of linearity for age was evaluated using restricted cubic splines (RCS), with four knots placed at the 5th, 35th, 65th, and 95th percentiles of the observed age distribution (8, 40, 61, and 86 years, respectively). In models where evidence of non-linearity was identified, the variable was retained in spline form. Cases with missing data on any covariates used in the models were removed from the analysis; thus, the number of included cases in each model depended on data availability.

Statistical significance was two-sided, and α = 0.05 was considered a significant threshold.

### 2.4. Ethical Considerations

This project was approved by the ethics committee of the County Emergency Hospital “Sf. Spiridon” Iasi, Romania (number 75 from 15 May 2026). Given the retrospective nature of the study, the need for informed consent was waived. The research was conducted in accordance with the principles of the Declaration of Helsinki.

## 3. Results

**Figure 1:**
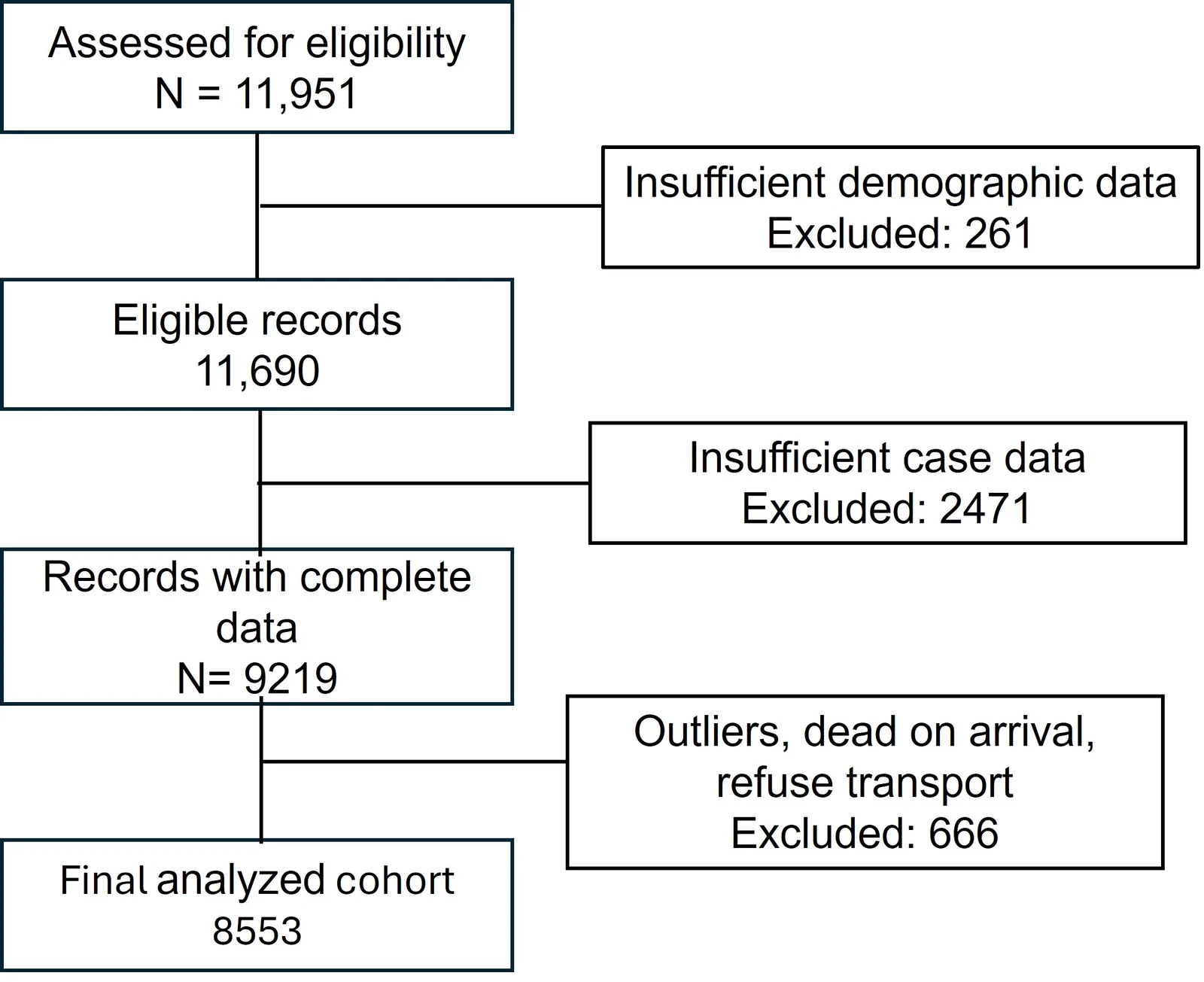
Study population selection flowchart.

Between January 2025 and February 2026, 11,951 trauma cases were identified in our database. Of these, 261 presented insufficient demographic data, and 2471 had missing case-specific data. Another 666 cases were removed either because the patients refused transport to the hospital, were dead on arrival, or were considered extreme outliers (Figure 1). The final cohort consisted of 8553 cases (61.9% male) with a relatively equal distribution between rural and urban areas (49.4% and 50.6%, respectively). Comparative analysis (Supplementary Table S2) highlights that the excluded cohort differed significantly from the included cohort regarding gender distribution, age, trauma type, triage severity, type of team dispatched, number of on-scene procedures, presence of polytrauma, and involvement in incidents with multiple victims (*p* < 0.001 for all). The removed cases were younger, male, were more often victims of a car crash, burn, or electrocution, and violence, and were more frequently classified as severe (red triage designation). These systematic differences between included and excluded cases may have introduced selection bias, resulting in a final cohort that was older and had lower trauma severity than the original dataset.

**Figure 2:**
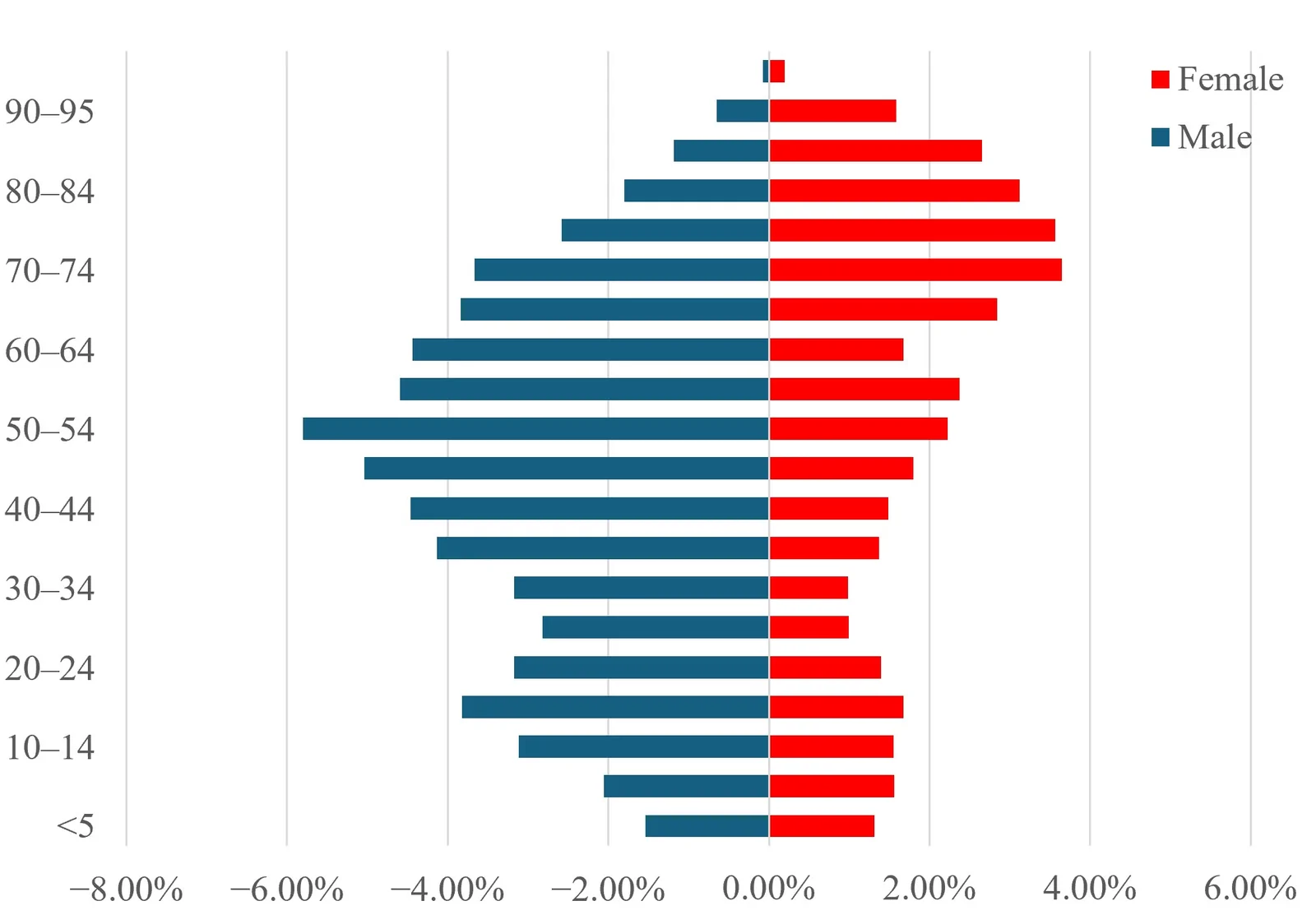
Age distribution of trauma cases according to gender.

Continuous variables in our sample exhibited non-Gaussian distributions, as indicated by the Kolmogorov–Smirnov test (*p* < 0.001 for all). The median age of the sample was 50 (IQR:30–69) years (15.7% underage under 18 years old and 31.4% elderly, over 65 years), with female trauma victims being older (median 60, IQR:35–86 years for females compared to 47, IQR:27–62 years for men, *p* < 0.001). Age distribution by gender is shown in Figure 2. A significant difference was identified between genders regarding the type of trauma, with more females presenting for falls (66.1% of females versus 57.5% of males, *p* < 0.001), while men were more likely to present for accidental trauma of head and neck (13.4% of men versus 11.2% of females, *p* = 0.003), and as result of violence (12.8% of men compared to 6% of women, *p* < 0.001). More male patients were considered severe (red) during triage (7.4% versus 4.5%, *p* < 0.001), and presented more often as a polytrauma case (3.2% versus 1.4%, *p* < 0.001). Trauma cases were relatively evenly distributed throughout the week, with approximately 14% of cases identified each day, and most occurred during the day (80.9%). Again, a significant difference was identified for gender, with more men presenting for trauma during weekends (30.1% versus 26.8%, *p* = 0.001) and during the night (21% versus 16%, *p* < 0.001). A multiple-victim incident was reported in 77 cases with equal distribution between genders. Other sample characteristics are presented in Table 1.

### 3.1. On-Scene Time

Median on-scene time in our sample was 16 min (IQR: 11–23), and 2815 (33.1%) interventions exceeded the 20 min cut-off. Longer operational intervals were identified for this metric for females (16 min, IQR:11–22 for males versus 17, IQR:11–24 for females, *p* < 0.001), the elderly (15 min, IQR:10–21, versus 19, IQR:14–26 for patients over the age of 65, *p* < 0.001), rural interventions (15, IQR:10–22 in urban versus 18, IQR:12–25 in rural, *p* <0.001), and for trauma occurring during the day (15, IQR: during the night versus 17, IQR: during the day, *p* < 0.001). Expectedly more severe trauma (20 min, IQR:15–28 for red triage designation versus 16 min, IQR:11–23 for yellow), such as car crashes (median 18 min, IQR:13–26), burns (17, IQR:10–24), drownings (25, IQR:19.75–55.5), and hypothermia-associated trauma (18, IQR:12–24), or in cases with polytrauma (20, IQR:14–27 for polytrauma versus 16, IQR:11–23), multiple victims (25, IQR:19–34 for multiple victim incidents versus 16, IQR:11–23 without), or when extrication was needed (31, IQR:20–39.5 for entrapped victims versus 16, IQR:11–23 without extrication) presented with higher on-scene time (*p* < 0.001 for each). Spearman’s correlation further showed that age and the number of interventions performed were positively correlated with on-scene time (r_s_ = 0.27 and 0.19, respectively; *p* < 0.001).

**Figure 3:**
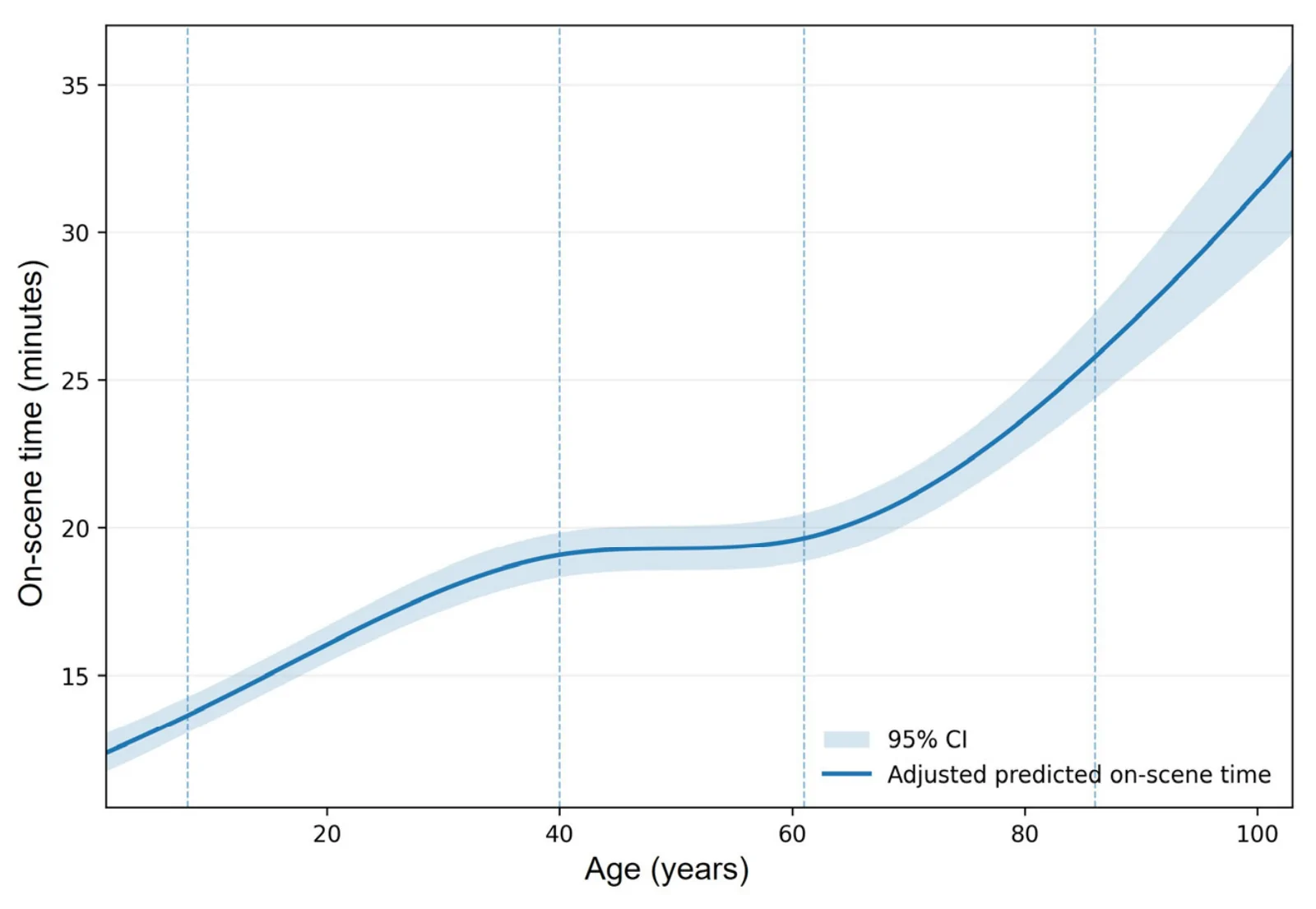
Adjusted association between age and on-scene time. Plotted estimates based on the primary generalized linear model with age modeled using restricted cubic splines with four knots placed at the 5th, 35th, 65th, and 95th percentiles of the observed age distribution (8, 40, 61, and 86 years, respectively). Shaded areas represent confidence intervals derived from model estimates. Predictions were plotted with all other covariates set at their reference category. The model shows a plateau tendency toward 20 min at ages between 40 and 60 years old.

Age showed evidence of non-linear association with on-scene time (likelihood-ratio test for non-linearity: χ^2^ = 68.60, df = 2, *p* < 0.001). All spline components were significant in our primary GLM, and modeling using splines improved model fit substantially (ΔAIC = −64.6). Consequently, age was modeled using restricted cubic splines for this outcome. The relationship between age and on-scene time is modeled in Figure 3.

**Figure 4:**
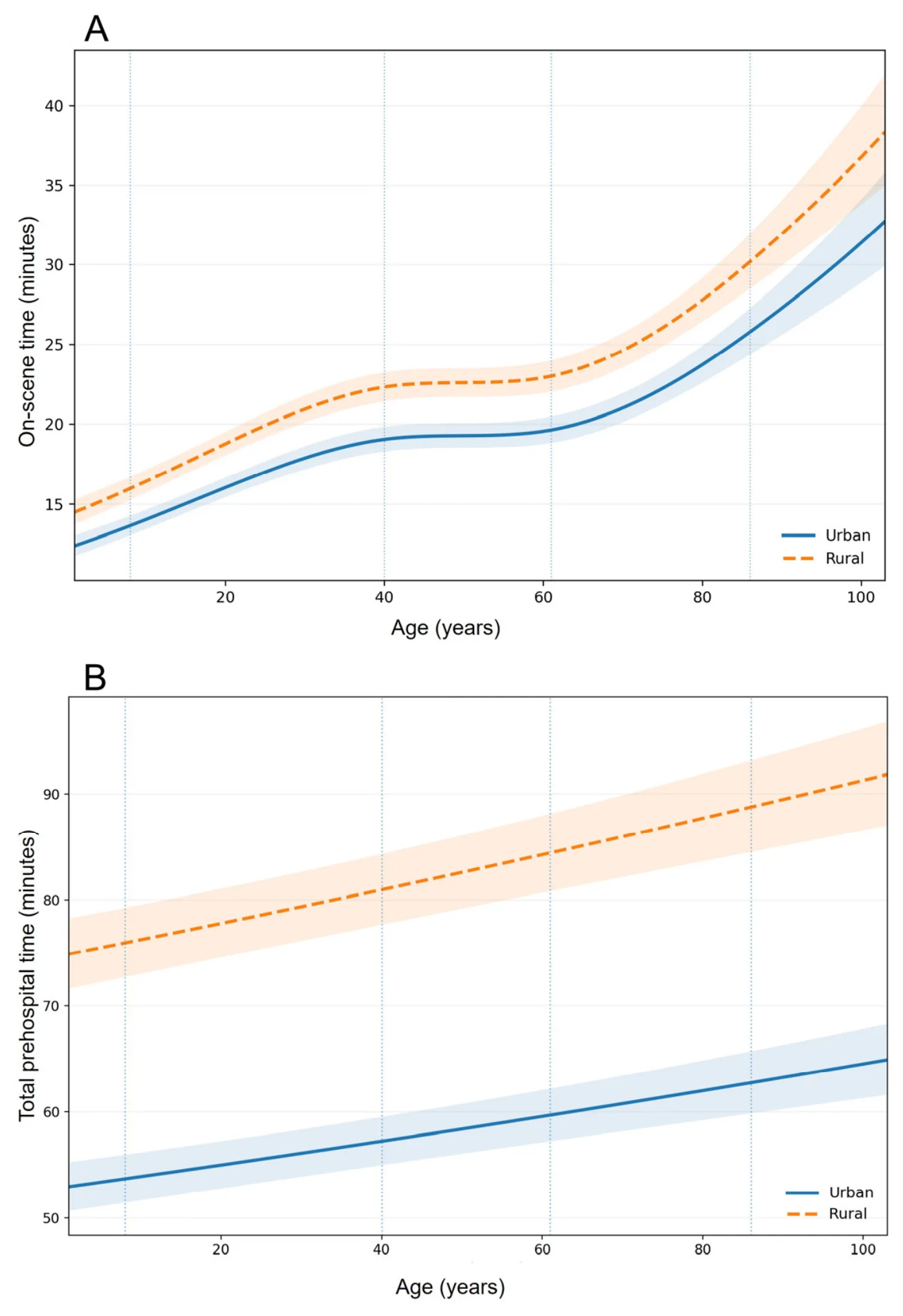
Adjusted predicted time for on-scene (**A**) and total prehospital time (**B**) plotted according to age and stratified according to environment (rural vs. urban). Predictions were plotted based on primary generalized linear model estimates for the two outcome variables. For on-scene time (A), age was modeled using restricted cubic splines with four knots placed at the 5th, 35th, 65th, and 95th percentiles of the observed age distribution (8, 40, 61, and 86 years, respectively). Shaded areas represent 95% CI. All other model variables were set at their reference category.

The primary adjusted GLM (Table 2) showed that age was non-linearly associated with on-scene time and that rural environment (TR = 1.17, 95%CI:1.15–1.2, *p* < 0.001) and greater trauma severity were associated with longer on-scene time (TR = 1.27, 95% CI:1.14–1.41, *p* < 0.001 for red triage designation and TR = 1.12, 95% CI:1.06–1.18, *p* < 0.001). Type of trauma was also significantly associated overall, and compared to car crashes, fires and explosions (marginal significance), falls, hypothermia, violence, and other trauma displayed decreased time ratios. The model shows an adjusted on-scene time that is 17% longer in rural environments. A comparison of the adjusted model that considers only age and environment is shown in Figure 4. Further sensitivity analysis was conducted by adjusting for scene complexity, such as extrication, multiple victims, and the number of interventions performed on scene (Supplementary Table S3). Direction and magnitude of the associations were retained for the variables included in the primary model, supporting the robustness of the main findings. This further analysis also highlighted the association of on-scene time with on-scene complexity, as time ratios were higher when multiple victims were involved (TR = 1.19, 95%CI:1.05–1.34, *p* = 0.005) and when more treatments were carried out on-scene (TR = 1.08, 95% CI:1.06–1.09, *p* < 0.001) but not when extrication was performed (TR = 1.15, 95% CI:0.8–1.64, *p* = 0.45).

When considering the clinically significant threshold of 20 min or more, the adjusted primary logistic regression identified age, rural environment, trauma type, and severity as factors independently associated with on-scene delays. Rural interventions had 74% higher adjusted odds of on-scene time delay compared with urban environments (OR = 1.74, 95% CI: 1.58–1.92, *p* < 0.001). Adjusted predicted probabilities for age in rural and urban environments for on-scene time ≥20 min and total prehospital time ≥60 min are plotted in Supplementary Figure S1. Other relevant factors identified were polytrauma (OR = 1.68, 95% CI: 1.35–2.08, *p* < 0.001) and trauma severity (OR = 2.5, 95% CI: 1.61–3.89, *p* < 0.001). No relevant multicollinearity between predictors was detected (VIFs up to 1.8, except for age splines, which is interpretable as a correlation between functions derived from the same variable and is expected). The Hosmer–Lemeshow test did not indicate evidence of lack of fit (HL test *p* = 0.08). ROC curve analysis showed modest discrimination (AUC = 0.679, 95% CI:0.667–0.691, *p* < 0.001). Apparent calibration yielded an intercept of 0 and a slope of 1, as expected from model testing on the development sample. The number of events-per-variable (EPV) in our sampled data was 141 for this model, supporting the robustness of the analysis. Further bootstrap resampling supported the stability of most major associations, showing minimal bias for the main predictors used in the analyses, although instability was observed for variables with few observations.

### 3.2. Total Prehospital Time

Total prehospital time exceeded 60 min in 59.5% of cases. Median total prehospital time in our sample was 67 (IQR: 44–99). This was found to be longer in female and elderly patients, during the weekdays, and specifically during daytime, and in patients who suffered burns, falls, or were victims of violence (*p* < 0.001 each). Interestingly, shorter total prehospital time was recorded for patients with severe triage designation (47, IQR: 35–69, for red versus 69, IQR: 45–101 for yellow, *p* < 0.001) and for intervention with multiple victims (60, IQR: 39–77 for multiple victims versus 67, IQR: 44–100 without, *p* = 0.008). Patients who presented with on-scene delays were more likely to have total prehospital time delays (*p* < 0.001); however, on-scene time was only weakly correlated with total prehospital time (R = 0.15, *p* < 0.001). For this time interval, Spearman’s correlation showed a weak positive correlation with age (r_s_ = 0.14, *p* < 0.001) and an inverse correlation with the number of interventions performed on-scene (r_s_ = −0.56, *p* < 0.001).

Restricted cubic spline analysis provided no evidence of a non-linear association between age and total prehospital time (χ^2^ = 1.6, *p* = 0.45); thus, it was retained in its linear form in further modeling. GLM for this outcome showed increased adjusted time ratios for age, rural environment, most trauma types, and severe triage designation (Table 3). These results were also retained following sensitivity analyses (Supplementary Table S4).

When considering a 60 min cut-off, adjusted logistic regression found a broadly similar pattern of factors associated with delayed total prehospital time. Interestingly, the direction of association with the helicopter rescue team was inverted. However, this must be interpreted with caution given the small number of events in this category. Again, model diagnostics showed a good fit, modest discriminative power (AUC = 0.742, 95% CI: 0.731–0.752, *p* < 0.001), and low risk of bias in bootstrapping for most included variables. EPV was 281 for this model. Apparent calibration also yielded an intercept of 0 and a slope of 1, as expected in the development sample.

## 4. Discussion

In this study, we explored factors associated with operational delays for on-scene and total prehospital time. Our findings suggest the importance of age and environment along with trauma-related characteristics for increased operational times. Trauma scene complexity also contributes to operational delays; however, the observed associations within our primary models remain similar in our sensitivity analysis. Among the most prominent findings of our study is the significant variability in prehospital trauma care operational time intervals with respect to age and rural environments. Our data show markedly increased operational times in this context, which warrant further exploration. The non-linear association between age and on-scene time is also interesting, suggesting that this operational time interval may increase in patients aged above 60 years. Overall, the prehospital time in our study was suboptimal for more than half of trauma responses, and on-scene operational time exceeded 20 min in up to a third of trauma cases, highlighting potential for further improvement and underscoring the importance of this assessment.

Previous research in Europe has also highlighted the importance of the environment in increasing prehospital intervention time for trauma patients. These data originate mainly from Scandinavian countries such as Norway and Sweden, where rural settings were found to have longer prehospital times [7,14]. However, these studies did not consider operational complexity and did not adjust for demographic and case-specific variables. We show that the rural environment is an important factor for both on-scene and total prehospital delays, even when adjusting for other factors. Not surprisingly, managing trauma in rural environments is emerging as an important contemporary issue of modern trauma systems, regardless of the emergency care system [21]. Trauma patients from rural environments have been found to experience worse outcomes and a higher risk of mortality [22]. This is in part due to operational challenges such as distance to nearby hospitals, lack of access to level I trauma centers, and need for secondary transfer [23]. However, these variables were not available in our dataset, and we could not adjust for these in our model. Other data show that rural interventions benefit more sparsely from physician-led teams and follow task-sharing models of care [24]. This was found in Norway, for instance, where remote areas were less likely to benefit from a physician-led team [25]. In our sample, response team type was not associated with the operational outcomes studied, except for helicopter rescue teams. Why rural interventions were associated with more on-scene delays in our sample remains unclear and warrants further investigation. However, given the large rural population in our settings, a deep understanding of possible factors affecting on-scene and total prehospital operational time is paramount. Potential strategies in need of further evaluation include rural-specific trauma pathways, early activation of trauma teams via dispatch, and streamlining decision-making regarding transport designation. Telemedicine support has also been proposed as a potential strategy to improve on-scene operations and may reduce delays, but it remains largely unexplored globally [26,27].

Our data show the importance of age as a possible factor for higher operational delays, particularly in rural settings. This is important as the rural population of Romania has a predominantly elderly population [28]. Older age has been found to be an important factor for prolonged intervention in trauma cases in several other studies [12,29]. Consequently, this is not a factor specific to our demographic. Still, this raises concerns as elderly patients are more vulnerable and face worse outcomes even in low-energy trauma [30]. This stems from inherent vulnerability due to preexisting conditions and diminished physiologic reserves, which predispose elderly individuals to adverse outcomes. Yet, studies show that elderly patients face undertriage that exposes them to inappropriate hospital admission and delayed trauma care, which adds further strain to this already vulnerable segment of the population with trauma [31,32]. This has prompted the emergence of specific care protocols for elderly individuals [33]. However, these focus mostly on in-hospital management and do not address the critical prehospital operational time. The reasons for these operational delays among geriatric patients in the prehospital phase remain unclear; as such, only limited actionable changes have been recommended for this critical period. This includes a reassessment of the in-the-field triage system based on physiological criteria and mechanism [34]. These protocols are in their early stages of development and have yet to prove their significance regarding patient outcomes. Other studies that have identified age as an important factor in operational delays suggest that unconscious cognitive bias in care patterns for elderly individuals may be a possible culprit [12,35]. Studies showed that elderly patients with severe trauma are less likely to be transported to a level I trauma center [36]. Age-related bias is an emergent concern within most trauma systems, an issue that has not yet been properly addressed [37]. These observations support the need to improve awareness of age-related challenges in prehospital trauma care and for further research on potentially modifiable factors among the elderly. Our study design did not allow us to investigate age-related bias. Further studies are needed to identify the underlying mechanisms of the observed association in our settings.

Finally, our analysis, including on-scene complexity, found an association between multiple victims and the number of on-scene treatment and operational times. However, this association must be interpreted carefully. The analysis did not account for the clinical indication for these treatments or for transport time. Also, no causal interpretation based on our models is possible. The discussion of whether to act or not on the scene remains debatable [18]. Some studies suggested that prolonged on-scene time and specific interventions may increase mortality [10]. We could not investigate the impact on in-hospital outcomes given our study design.

The present study has as elements of originality the consideration of a wide range of factors, including previously unexplored operational complexity factors, while examining individual components of the prehospital phase of trauma care. This study also offers a characterization of prehospital trauma care in a significant geographical region of Eastern Europe with a developing emergency care system. However, this study presents several limitations. The present study was designed to evaluate operational factors associated with increased prehospital times in trauma rather than patient-centered clinical outcomes, thereby limiting the relevance of the study findings. It was conducted in a single county, which limits generalizability. The data were retrospectively collected, which relies on the accuracy of the dispatch registry and may be subject to collection bias. A consistent proportion of entries were removed due to incomplete demographic and operational data. We noted that the excluded cases differed systematically from the analyzed cohort across demographic and trauma-related characteristics. Therefore, selection bias cannot be excluded, and the selected cohort may have been older and had lower trauma severity than the population from which it was drawn. Several potential factors were not available in our database. These include distance to the scene and nearest hospital, traffic and weather conditions, travel time, hospital destination, and response team availability, which might represent residual confounding. Also, trauma severity was captured using the START triage code, which does not reflect actual injury severity, limiting any conclusion drawn regarding the severity of trauma. Some subgroups in our analysis presented a limited number of cases, such as drowning, extrication, or teams involving mobile intensive care units, and results regarding these must be interpreted with caution. Finally, this study captures only a limited time frame and cannot ascertain the deterministic effect of these variables studied, as this requires a prospective design.

## 5. Conclusions

Total prehospital time in our study exceeded the predefined clinically relevant threshold of 60 min in more than half of patients, while up to a third exceeded the 20 min on-scene time threshold. This supports the need to explore modifiable factors for both on-scene and total prehospital delays in trauma care. Our study observations show that operational times are associated not only with trauma and scene-related characteristics, but also demographic and environmental factors. Particularly, age and rural environment were associated with both outcomes. Further studies are needed to understand the operational challenges in older trauma patients and to highlight specific strategies to improve operational efficiency in rural settings.

## Figures and Tables

**Table 1:** Sample characteristics.

Variable	
Gender Male Nr (%)	5274 (61.9%)
Age, years Median (IQR)	50 (30–69)
Urban Environment Nr (%)	4305 (50.6%)
Moment of intervention	
Workday daytime Nr (%)	4965 (58.3%)
Workday nighttime Nr (%)	1091 (12.8%)
Weekend daytime Nr (%)	1924 (22.6%)
Weekend nighttime Nr (%)	535 (6.3%)
Trauma type	
Car crash Nr (%)	1049 (12.3%)
Burns and electrocutions Nr (%)	219 (2.6%)
Falls Nr (%)	5172 (60.7%)
Hypothermia and trauma Nr (%)	127 (1.5%)
Drowning and trauma Nr (%)	6 (0.1%)
Violence Nr (%)	869 (10.2%)
Other trauma Nr (%)	1071 (12.6%)
START triage code	
Yellow (delayed) Nr (%)	7979 (93.7%)
Red (immediate) Nr (%)	536 (6.3%)
Rescue team	
Nurse-led Emergency Medical Unit Nr (%)	8019 (94.2%)
Physician-led Emergency Medical Unit Nr (%)	441 (5.2%)
Paramedic-led Emergency Medical Unit Nr (%)	22 (0.3%)
Helicopter rescue medical team Nr (%)	27 (0.3%)
Mobile intensive care unit Nr (%)	4 (0.0001%)
Number of on-site interventions	
0 Nr (%)	5746 (67.5%)
1 Nr (%)	1051 (12.3%)
2 Nr (%)	1459 (17.1%)
3 Nr (%)	202 (2.4%)
4 or more Nr (%)	57 (0.7%)
Extrication Nr (%)	8 (0.01%)
Multiple victims Nr (%)	77 (0.9%)
Polytrauma Nr (%)	386 (4.5%)
Total	8553

**Table 2:** Multivariable analyses of factors associated with on-scene time.

	Generalized Linear Model		Multivariable Logistic Regression for On-Scene Time ≥20 min	
	TR (95%CI)	*p*	OR (95%CI)	*p*
Age		<0.001 *		<0.001 *
Gender (Female)	1.02 (1–1.05)	0.08	1.08 (0.97–1.19)	0.16
Environment (Rural)	1.17 (1.15–1.2)	<0.001	1.74 (1.58–1.92)	<0.001
Moment of intervention				
Workday daytime	Ref.	Ref.	Ref.	Ref.
Workday nighttime	0.94 (0.91–0.97)	<0.001	0.8 (0.69–0.94)	0.006
Weekend daytime	0.99 (0.96–1.02)	0.37	1.01 (0.9–1.13)	0.89
Weekend nighttime	0.96 (0.92–1.01)	0.08	0.85 (0.69–1.05)	0.13
Trauma type				
Car crash	Ref.	Ref.	Ref.	Ref.
Fires and explosions	0.93 (0.86–1)	0.058	0.73 (0.52–1.01)	0.054
Falls	0.83 (0.8–0.86)	<0.001	0.53 (0.46–0.62)	<0.001
Hypothermia	0.88 (0.8–0.97)	0.01	0.56 (0.38–0.84)	0.005
Drowning	1.3 (0.86–1.96)	0.22	5.93 (0.52–67.89)	0.15
Violence	0.89 (0.84–0.93)	<0.001	0.59 (0.48–0.72)	<0.001
Other trauma	0.83 (0.79–0.86)	<0.001	0.48 (0.4–0.59)	<0.001
START triage code (Red)	1.27 (1.14–1.41)	<0.001	2.5 (1.61–3.89)	<0.001
Rescue team				
Nurse-led Emergency Medical Unit	Ref.	Ref.	Ref.	Ref.
Physician-led Emergency Medical Unit	1.01 (0.9–1.13)	0.92	0.83 (0.51–1.33)	0.44
Paramedic-led Emergency Medical Unit	0.87 (0.7–1.08)	0.20	0.48 (0.16–1.49)	0.20
Helicopter rescue medical team	0.87 (0.72–1.06)	0.16	0.6 (0.23–1.56)	0.30
Mobile intensive care unit	1.27 (0.76–2.12)	0.36	1.73 (0.17–17.69)	0.64
Polytrauma	1.12 (1.06–1.18)	0.001	1.68 (1.35–2.08)	<0.001

* Modeled using restricted cubic splines with four knots (5th, 35th, 65th, and 95th percentiles). Because the association was non-linear, a single time ratio coefficient is not reported.

**Table 3:** Multivariable analyses of factors associated with total prehospital time.

	Generalized Linear Model		Multivariable Logistic Regression for Total Prehospital Time > 60 min	
	TR (95%CI)	*p*	OR (95%CI)	*p*
Age	1.0024 (1.0019–1.0029)	<0.001	1.008 (1.006–1.01)	<0.001
Gender (Female)	1.01 (0.98–1.03)	0.70	0.96 (0.87–1.06)	0.42
Environment (Rural)	1.42 (1.38–1.45)	<0.001	4.91 (4.45–5.43)	<0.001
Moment of intervention				
Workday daytime	Ref.	Ref.	Ref.	Ref.
Workday nighttime	0.72 (0.7–0.75)	<0.001	0.52 (0.45–0.6)	<0.001
Weekend daytime	0.84 (0.82–0.86)	<0.001	0.66 (0.58–0.74)	<0.001
Weekend nighttime	0.75 (0.72–0.79)	<0.001	0.52 (0.43–0.63)	<0.001
Trauma type				
Car crash	Ref.	Ref.		
Fires and explosions	1.37 (1.26–1.48)	<0.001	3.16 (2.24–4.46)	<0.001
Falls	1.31 (1.26–1.36)	<0.001	2 (1.71–2.33)	<0.001
Hypothermia	0.97 (0.88–1.08)	0.59	0.98 (0.65–1.47)	0.90
Drowning	1.21 (0.67–2.2)	0.53	3.26 (0.51–20.92)	0.21
Violence	1.26 (1.2–1.33)	<0.001	1.8 (1.47–2.22)	<0.001
Other trauma	1.15 (1.09–1.2)	<0.001	1.31 (1.08–1.59)	0.006
START triage code (Red)	0.77 (0.68–0.87)	<0.001	0.56 (0.36–0.87)	0.009
Rescue team				
Nurse-led Emergency Medical Unit	Ref.	Ref.	Ref.	Ref.
Physician-led Emergency Medical Unit	1.05 (0.92–1.19)	0.51	0.75 (0.46–1.21)	0.24
Paramedic-led Emergency Medical Unit	1.04 (0.83–1.29)	0.75	1.53 (0.59–3.93)	0.38
Helicopter rescue medical team	0.20 (0.02–0.36)	0.03	3.52 (1.15–10.77)	0.03
Mobile intensive care unit	0.84 (0.4–1.77)	0.65	0.63 (0.07–5.72)	0.68
Polytrauma	1.02 (0.96–1.07)	0.58	0.9 (0.72–1.14)	0.40

## Data Availability

Data used in this study are available at https://doi.org/10.5281/zenodo.20488362.

## Supplementary Materials

The following supporting information can be downloaded at: https://www.mdpi.com/article/10.3390/medicina62091632/s1.

## Abbreviations

The following abbreviations are used in this manuscript:

EMS	Emergency Medical System
SMURD	Mobile Emergency Service for Resuscitation and Extrication
STAR	Simple Triage and Rapid Treatment
VIF	Variance Inflation Factors
IQR	Interquartile Range
