# Supplementary material for: Factors Influencing Operational Delays in Prehospital Trauma Interventions: Evidence from a Romanian Cohort
Source: Medicina (Kaunas). 2026 Aug 25;62(9):1632. doi: 10.3390/medicina62091632 (PMC13608640; doi:10.3390/medicina62091632)
Supplement: Supplementary file 1 [file medicina-62-01632-s001.zip › Supplementary Table S2.pdf]

Supplementary Table S2. Comparison of demographic and trauma characteristics between included and excluded cases.

Variable	Included cohort	Excluded cohort	P
Gender Male Nr(% of category)	5,274(61.9%)	2074(61.0%)	<0.001
Age Median(IQR)	50(30–69)	46(26-64)	<0.001
Urban Environment Nr(% of category)	4305(50.6%)	1730(50.9%)	0.32
Moment of intervention			0.16
Workday daytime Nr(% of category)	4965(58.3%)	2370(69.7%)	
Workday nighttime Nr(% of category)	1091(12.8%)	632(18.6%)	
Weekend daytime Nr(% of category)	1924(22.6%)	298(8.7%)	
Weekend nighttime Nr(% of category)	535(6.3%)	59(1.8%)	
Trauma type			<0.001
Car crash Nr(% of category)	1049(12.3%)	1007(29.6%)	
Burns and electrocutions Nr(% of category)	219(2.6%)	143(4.2%)	
Falls Nr(% of category)	5172(60.7%)	1294(38.1%)	
Hypothermia and trauma Nr(% of category)	127 (1.5%)	37(1%)	
Drowning and trauma Nr(% of category)	6(0.1%)	2(0.05%)	
Violence Nr(% of category)	869(10.2%)	487(14.3%)	
Other trauma Nr(% of category)	1071(12.6%)	389(11.4%)	
START triage code			<0.001
Yellow (delayed) Nr(% of category)	7,979(93.7%)	3041(98.4%)	
Red (immediate) Nr(% of category)	536(6.3%)	318(9.4%)	
Rescue team			<0.001
Nurse-led Emergency Medical Unit Nr(% of category)	8019(94.2%)	2751(80.9%)	
Physician-led Emergency Medical Unit Nr(% of category)	441(5.2%)	237(6.9%)	
Paramedic-led Emergency Medical Unit Nr(% of category)	22(0.3%)	302(8.9%)	
Helicopter rescue medical team Nr(% of category)	27(0.3%)	30(0.8%)	
Mobile intensive care unit Nr(% of category)	4(0.0001%)	24((0.7%)	
Number of on-site interventions			<0.001
0 Nr(% of category)	5746(67.5%)	2849(83.8%)	
1 Nr(% of category)	1051(12.3%)	196(5.7%)	
2 Nr(% of category)	1459(17.1%)	253(7.4%)	
3 Nr(% of category)	202(2.4%)	50(1.4%)	
4 or more Nr(% of category)	57(0.7%)	11(0.3%)	
Extrication Nr(% of category)	8(0.01%)	5(0.1%)	0.41
Multiple victims Nr(% of category)	77(0.9%)	99(2.9%)	<0.001
Polytrauma Nr(% of category)	386(4.5%)	95(2.8%)	<0.001
Total	8,553	3398	
