# Supplementary material for: Factors Influencing Operational Delays in Prehospital Trauma Interventions: Evidence from a Romanian Cohort
Source: Medicina (Kaunas). 2026 Aug 25;62(9):1632. doi: 10.3390/medicina62091632 (PMC13608640; doi:10.3390/medicina62091632)
Supplement: Supplementary file 1 [file medicina-62-01632-s001.zip › Supplementary Table S3.pdf]

Supplementary Table S3. Sensitivity analysis for adjusted multivariable analysis of on-scene time

	**Generalized linear model**		**Multivariable logistic regression for on-scene time ≥20 minutes**	
	TR(95%CI)	P	OR(95%CI)	P
Age		<0.001*		<0.001*
Gender (Female)	1.02(1–1.05)	0.04	1.09(0.98–1.21)	0.10
Environment (Rural)	1.16(1.13–1.18)	<0.001	1.66(1.51–1.84)	<0.001
Moment of intervention				
Workday daytime	Ref.	Ref.	Ref.	Ref.
Workday nighttime	0.94(0.91–0.97)	0.001	0.82(0.7–0.96)	0.01
Weekend daytime	0.99(0.96–1.01)	0.26	1(0.89–1.13)	0.94
Weekend nighttime	0.96(0.92–1.01)	0.12	0.88(0.71–1.08)	0.23
Trauma type				
Car crash	Ref.	Ref.	Ref.	Ref.
Fires and explosions	1.02(0.95–1.1)	0.62	1(0.72–1.4)	0.99
Falls	0.88(0.85–0.92)	<0.001	0.67(0.57–0.79)	<0.001
Hypothermia	0.97(0.88–1.07)	0.54	0.8(0.53–1.2)	0.28
Drowning	1.37(0.91–2.07)	0.13	7.77(0.7–85.9)	0.10
Violence	0.95(0.91–1)	0.05	0.76(0.62–0.95)	0.01
Other trauma	0.87(0.83–0.91)	<0.001	0.59(0.48–0.72)	<0.001
START triage code (Red)	1.19(1.07–1.32)	0.002	2.02(1.29–3.17)	0.002
Rescue team				
Nurse-led Emergency Medical Unit	Ref.	Ref.	Ref.	Ref.
Physician-led Emergency Medical Unit	1.03(0.92–1.16)	0.56	0.89(0.55–1.44)	0.63
Paramedic-led Emergency Medical Unit	0.92(0.74–1.14)	0.44	0.6(0.2–1.83)	0.37
Helicopter rescue medical team	0.87(0.72–1.06)	0.16	0.61(0.24–1.6)	0.32
Mobile intensive care unit	1.42(0.85–2.36)	0.18	2.38(0.23–25.26)	0.47
Number of on-site interventions	1.08(1.06–1.09)	<0.001	1.31(1.23–1.38)	<0.001
Multiple victims	1.19(1.05–1.34)	0.005	2.21(1.3–3.76)	0.003

Polytrauma	1.06(1.01–1.12)	0.03	1.41(1.13–1.76)	0.003
Extrication	1.15(0.8–1.64)	0.45	0.99(0.21–4.68)	0.99

* modeled using restricted cubic splines with four knots (5th, 35th, 65th, and 95th percentiles). Because the association was non-linear, a single Time Ratio coefficient is not reported.
