# Supplementary material for: Factors Influencing Operational Delays in Prehospital Trauma Interventions: Evidence from a Romanian Cohort
Source: Medicina (Kaunas). 2026 Aug 25;62(9):1632. doi: 10.3390/medicina62091632 (PMC13608640; doi:10.3390/medicina62091632)
Supplement: Supplementary file 1 [file medicina-62-01632-s001.zip › Supplementary Table S4.pdf]

Supplementary Table S4. Sensitivity analysis for adjusted multivariable analysis of total prehospital time

	**Generalized linear model**		**Multivariable logistic regression for total prehospital time ≥60 minutes**	
	TR(95%CI)	P	OR(95%CI)	P
Age	1.0024(1.0019 1.0029)	<0.001	1.008(1.006–1.01)	<0.001
Gender (Female)	1.001(0.98–1.03)	0.95	0.95(0.86–1.05)	0.33
Environment (Rural)	1.43(1.39–1.46)	<0.001	5.04(4.56–5.58)	<0.001
Moment of intervention				
Workday daytime	Ref.	Ref.	Ref.	Ref.
Workday nighttime	0.75(0.71–0.79)	<0.001	0.51(0.44–0.59)	<0.001
Weekend daytime	0.84(0.82–0.87)	<0.001	0.65(0.58–0.74)	<0.001
Weekend nighttime	0.72(0.69–0.75)	<0.001	0.51(0.42–0.63)	<0.001
Trauma type				
Car crash	Ref.	Ref.	Ref.	Ref.
Fires and explosions	1.11(1.06–1.17)	<0.001	2.77(1.95–3.93)	<0.001
Falls	1.22(1.16–1.28)	<0.001	1.84(1.56–2.16)	<0.001
Hypothermia	1.2(0.66–2.18)	0.55	0.85(0.56–1.28)	0.43
Drowning	0.92(0.83–1.02)	0.12	2.98(0.47–19.05)	0.25
Violence	1.27(1.22–1.32)	<0.001	1.65(1.33–2.04)	<0.001
Other trauma	1.3(1.2–1.41)	<0.001	1.22(1–1.49)	0.051
START triage code (Red)	0.8(0.7–0.9)	<0.001	0.6(0.38–0.93)	0.02
Rescue team				
Nurse-led Emergency Medical Unit	Ref.	Ref.	Ref.	Ref.
Physician-led Emergency Medical Unit	0.78(0.37–1.63)	0.50	0.75(0.46–1.22)	0.25
Paramedic-led Emergency Medical Unit	1.04(0.83–1.29)	0.75	1.4(0.54–3.62)	0.49
Helicopter rescue medical team	0.72(0.54–0.96)	0.02	3.52(1.15–10.76)	0.03
Mobile intensive care unit	1.04(0.91–1.18)	0.61	0.5(0.06–4.49)	0.54
Number of on-site interventions	0.95 (0.94–0.97)	<0.001	0.86 (0.81–0.91)	<0.001
Multiple victims	0.98(0.87–1.12)	0.80	1(0.78–1.27)	0.97
Polytrauma	1.05(0.99–1.11)	0.11	1.22(0.74–2.01)	0.45
Extrication	1.21(0.82–1.79)	0.35	2.4(0.47–12.3)	0.29
