# Supplementary material for: Factors Influencing Operational Delays in Prehospital Trauma Interventions: Evidence from a Romanian Cohort
Source: Medicina (Kaunas). 2026 Aug 25;62(9):1632. doi: 10.3390/medicina62091632 (PMC13608640; doi:10.3390/medicina62091632)
Supplement: Supplementary file 1 [file medicina-62-01632-s001.zip › Supplementary Figure S1.pdf]

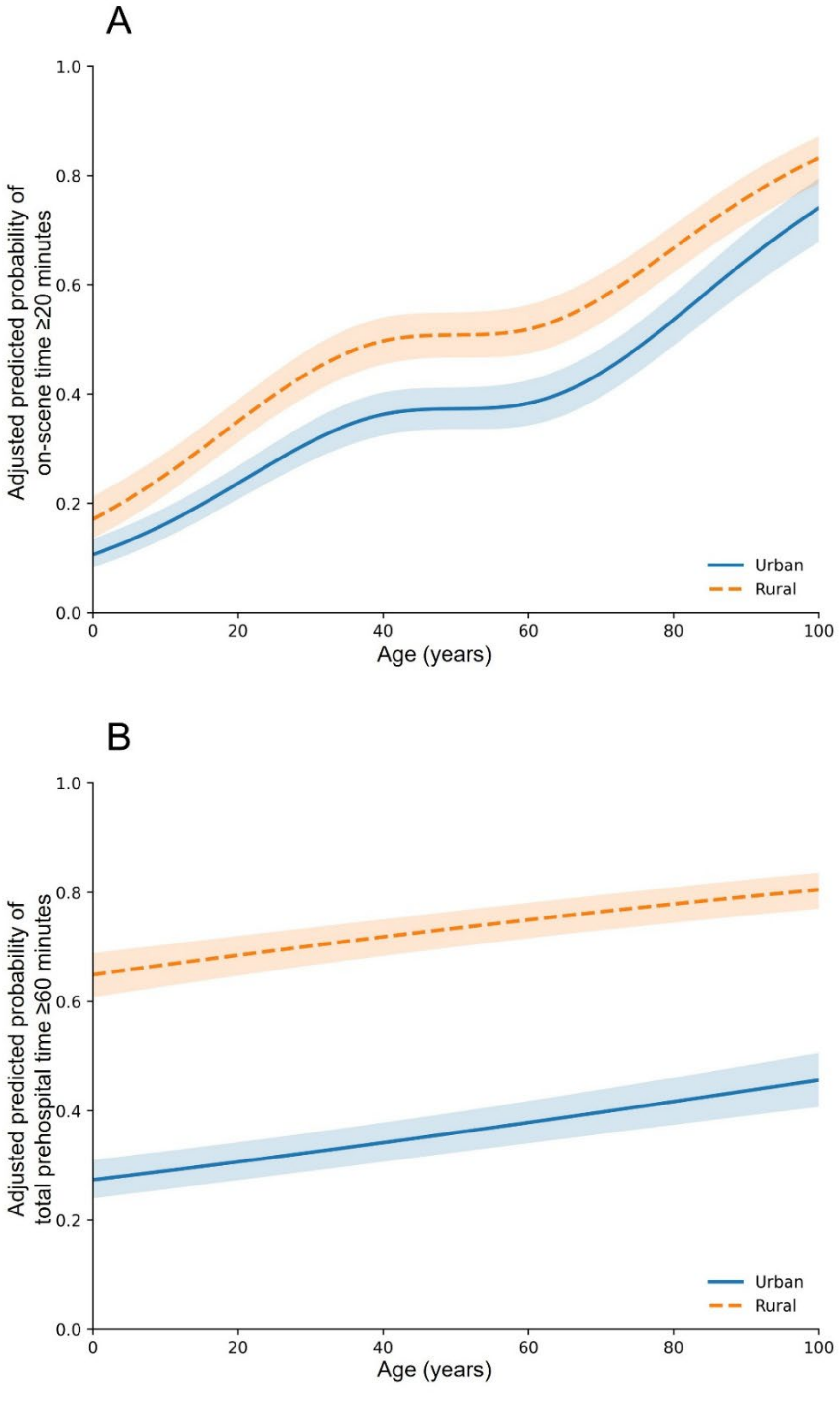

Supplementary Figure S1. Adjusted predicted probability for on-scene (A) and total prehospital time (B) plotted according to age and stratified according to environment (rural vs. urban). Predictions were plotted based on primary logistic model estimates for the two outcome variables. For on-scene time (A), age was modeled using restricted cubic splines with four knots placed at the 5th, 35th, 65th, and 95th percentiles of the observed age distribution (8, 40, 61, and 86 years, respectively). Shaded areas represent 95% CI. All other model variables were set at their reference category.
